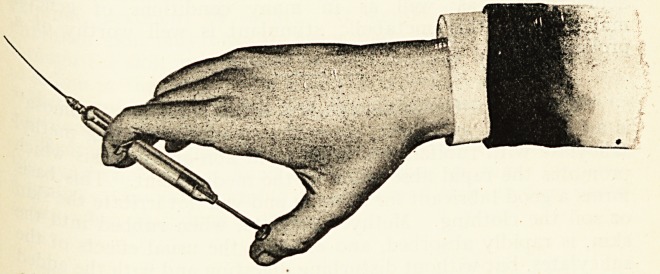# Notes on Preparations for the Sick

**Published:** 1912-09

**Authors:** 


### Notes on Preparations for the Sick.

"**Laibose.**" A concentrated food prepared from whole wheat and the solids of milk, to be taken with water as desired.—FAIRCHILD BROS. & FOSTER, New York. Agents: BURROUGHS, WELLCOME & CO., London.—The analytical reports issued with this preparation gives the protein content as 18 per cent., fat 17 per cent., carbohydrate 55 per cent. mineral ash 4 per cent.

Our examination showed the presence of those constituents which are always present in a food prepared from milk and wheat, and a certain proportion of starch was detected. A small portion of the food is soluble in water, but rather less than is usually the case.

---

"**Kerol.**"—QUIBELL BROTHERS LTD., Newark.—This is a name given to a series of disinfectant preparations which are now being much advertised. The bactericidal value of "Kerol" has been tested by reliable authorities using modern methods, and found quite satisfactory.

Judging from our analysis, "Kerol" is a tar product containing an emulsifying agent which enables it to mix quite readily with water, the resultant emulsion being fairly stable. Carbolic acid was not detected, and the reactions obtained indicate the presence of the higher congeners of phenol, which are less toxic but equally valuable as germicides.

---

"**Glaxo.**"—BRAND & CO., Vauxhall, S.W.—"Glaxo" is a starch-free infant's food, prepared by the old and reliable firm of Brand & Co. A notable feature is that the preparation is put on the market in hermetically-sealed tins, which is a most important point.

The food forms a milk-like emulsion, which we found to be rich in proteid. Soluble carbohydrate was present, but no starch was detected. It is one of the best milk foods that has come under our observation.

**Levico Water.**—Imported by HERTZ & Co., 9 Mincing Lane, London, E.C.—Levico Waters come from two springs situated near the town of Levico, in the Tyrol. One of the springs yields a strong, the other a mild water. We have examined a specimen of the former, and found it to be a genuine ferruginous mineral water. It possesses a distinctly astringent taste, and a faintly acid reaction.

The main constituent is ferrous sulphate, and small amounts of arsenic were detected, which enhance its tonic properties. We consider that ferrous iron possesses more valuable medicinal qualities than ferric compounds, and those taking this water can rely on a good proportion of the former combination being present.

---

**"Hyposol" Ampoule-Syringe.**—ALLEN & HANBURY'S LTD., London.—This combination supplies a sterile, solution, syringe and needle, always ready for immediate use. The ampoule becomes the barrel of the syringe, a piston rod being fitted on the one end and a finger-grip with needle at the other. The plunger is of pure cellulose, and fits the barrel perfectly. Sterilised needles are supplied, each enclosed in a sterile glass sheath.

The "Hyposol" can be supplied charged with any required solution up to 2 c.c. for subcutaneous, intra-muscular, or intra-spinal injections.

The ease with which the "Hyposol" is converted into a rigidly aseptic hypodermic syringe charged with a sterile solution is apparent, and its convenience as a substitute for the ordinary hypodermic syringe will easily be realised. For the complete success of the new method it is necessary that its cost should not appreciably exceed that of the old.

The expense of frequently renewing fragile syringes is not inconsiderable, and if this be taken into account, with the saving of time and the undoubted convenience, the extra cost per " Hyposol " above that of the ordinary ampoule is not considerable.

For insoluble mercurials, and other substances in suspension, the " Hyposol " method of administration is ideal.

---

**Malt Extract (crystalline) with Iron Iodide.**—A. WANDER LTD., 1–3 Leonard Street, London, E.C.—This is not a new preparation, but one which has been well tried. It is found that the mild laxative action of the malt extract corrects the constipating tendency of the iron, and that its action combines the properties of its components in a very efficient manner. The taste of the ferrous iodide is well disguised, and the combination is in every way an acceptable one.

---

**Glycerole Lecithin—Fairchild.**—BURROUGHS, WELLCOME & CO., London.—This solution of Lecithin contains 1 grain in each teaspoonful dose ; the disagreeable properties of the drug have been successfully disguised, and the Lecithin in solution is not coagulated by boiling or precipitated by alcohol. A dose contains gr. $\frac{1}{25}$ of actual phosphorus in its organic combination. In cases of cerebral exhaustion and the various forms of neurasthenia, as well as in many conditions of general malnutrition, this metabolic stimulant is well worthy of a prolonged trial.

---

"**Balmosa.**"—OPPENHEIMER, SON & CO., London.—"Balmosa" is an analgesic, antiseptic, snow-like cream, containing methyl salicylate with rubifacients in an ideal non-greasy basis which promotes the rapid absorption of the medicament. This basis forms a good lubricant for rubbing and will not irritate the skin or soil the clothing. Methyl salicylate, when rubbed into the skin, is rapidly absorbed, and exhibits the usual effects of the salicylates, but without disturbing digestion and with the added advantage that it can be applied in the neighbourhood of the pain.

---

**Palatinoid Caryophylli Pulv. gr. v—Keratine coated.**—OPPENHEIMER, SON & CO., London.—The therapeutic effects of cloves are chiefly due to the volatile oil present, but it is a fact that for internal use the powdered drug gives better results than the isolated oil. This may be due chiefly to mechanical causes, *e.g.* presence of fibre delaying absorption, but it is probable the other constituents—not present in the oil—also exert some action, hence the freshly-powdered drug is preferable for internal use, and it is best given in " Palatinoid " form, which preserves the volatile constituents perfectly, and the keratine coating, resisting solution by the gastric juice, permits the drug to pass the stomach unchanged, but is dissolved in the alkaline fluid in the intestines, thus liberating the contents where they can exert a maximum therapeutic effect.

---

**Sapo Antisepticus Etherius,** containing hyd. iodid. rub. 1 in 1,000. **Elixir pro Pertussi,** phenazonum, tolu and glycerine. **Vaginal Douche Tablets,** alum, zinc, sulpho-carbolat, etc. **Flexible Gelatine Capsules,** many varieties, *vide* list. **Asensitine,** a sterilised and antiseptic hypodermic solution of tropocain, 1 per cent. for dental anæsthesia.—DUNCAN, FLOCKHART & Co., Edinburgh.

These preparations are all the best of their kind, and their utility is obvious.

---

**Unguentum "Iothion," Bayer.**—THE BAYER CO., London. —A non-staining iodine ointment, containing 10 per cent. of "Iothion" (equivalent to 8 per cent. of iodine); is not combined with any alkali, but is prepared with a water-free ointment base. It has a slight yellow colour, with very little odour ; it diffuses easily, and should obviously be most useful for any form of external treatment by iodine. It should be applied to the unbroken skin only once or twice daily, when its absorption through the skin may readily be demonstrated by the presence of iodine in the urine. Its utility is especially obvious in the reduction of swollen glands and the disappearance of parasitic affections of the skin.

**Hydrastinin - hydrochlor, Bayer.** — In tablets containing the synthetic drug. Each contains 0.025 gramme (approx. $\frac{2}{5}$ grain). One tablet should be swallowed three or four times a day when this drug is indicated.

---

**Pituitary Extract : Vaporole.**—BURROUGHS, WELLCOME & Co., London.—Each phial contains 0.5 c.c. of a sterile extract made from the posterior lobe of the pituitary gland.

This product has the power of causing unstriped muscle to contract. It has proved especially useful in arresting the fall of blood-pressure in the condition of shock following surgical procedures. When injected into muscle the effect on the blood pressure is not noticeable until twenty or thirty minutes have elapsed, but an immediate result follows injection into a vein. In cases of shock the most convenient and efficient method of administering the extract is by adding it to normal saline solution, and transfusing intravenously with this mixture.

Its effect upon unstriped muscle is well seen in the action of the drug upon the uterus, for which reason it is now extensively used as a substitute for ergot.

Its action upon the intestinal muscles seems to be less constant, and more experience is required before one can confidently select cases favourable for its employment in intestinal paresis.

We have seen no ill effects following its use.

## Figures and Tables

**Figure f1:**